# Functional Outcomes Following Arthroscopic Anterior Cruciate Ligament (ACL) Reconstruction Using the Sironix Titanium Button and the Polyetheretherketone (PEEK) Button: A Retrospective Observational Study

**DOI:** 10.7759/cureus.46186

**Published:** 2023-09-29

**Authors:** Sunil K Dash, Dinesh Mishra, Harekrushna Sahu, Ashok K Moharana, Sachin Angrish, Deepak TS

**Affiliations:** 1 Orthopaedics, AMRI Hospitals, Bhubaneswar, IND; 2 Clinical Affairs, Healthium Medtech Limited, Bengaluru, IND

**Keywords:** surestitch, reconstruction surgery, proloop, peek button, anterior cruciate ligament

## Abstract

Background

Anterior cruciate ligament (ACL) injury is most common among athletes compared to the general population. ACL reconstruction is a clinical standard for restoring joint mechanical stability and enabling sports return. The purpose of the study is to evaluate the safety and functional outcomes after arthroscopic ACL reconstruction using the Sironix titanium button and the polyetheretherketone (PEEK) button.

Methods

A total of 31 subjects who have undergone arthroscopic ACL reconstruction using the Sironix titanium button and PEEK button between August 2022 and January 2023 were included in the study. Demographic data, surgery details, and other baseline characteristics of the subjects were collected from the hospital records. The primary objective of the study was to assess the functional outcome using the International Knee Documentation Committee (IKDC) questionnaire. The secondary objectives were to determine the pre- and post-surgery activity levels using the Tegner Activity Score (TAS) and Lysholm score. Quality of life evaluation was done by using the Quality of Life (QoL) subscale from the Knee Injury and Osteoarthritis Outcome Score (KOOS) and Single Assessment Numerical Evaluation (SANE). Device-related adverse effect information was recorded.

Results

The mean (SD) of the total IKDC score of 31 subjects at baseline and post-surgery was 51.4 (2.84) and 91.8 (2.59) out of 100, respectively. The mean (SD) of TAS pre-injury and post-surgery was 5.3 (1.47) and 5.4 (1.38) out of 10, respectively. The total mean (SD) value of the total Lysholm Score at baseline and post-surgery was 53.9 (3.72) and 91.4 (3.61) out of 100, respectively. The mean (SD) value of the quality of life subscale of the KOOS score was 91.2 (3.91) out of 100. The total mean (SD) value of the SANE score that had affected joint/region of interest today was 97.4 (1.78), while for the opposite side today, it was 99.5 (0.85) out of 100. There were no adverse device effects reported in this study.

Conclusion

Based on the score assessment, it was observed that the performance of Sironix knee implant devices, Proloop-Titanium adjustable loop button, T-Button A® Closed PEEK button, and Surestitch® All Inside Meniscal Repair Implant (Healthium Medtech Limited, Bengaluru, Karnataka, India) was effective and safe with no adverse effects. Therefore, Sironix knee implants are considered safe and effective in ACL reconstruction and meniscus repair surgery.

## Introduction

The anterior cruciate ligament (ACL) is one of the four major ligaments in the knee, which plays an essential role in joint stability during regular activities [1,2]. It is the most often injured ligament in the knee, frequently occurring in basketball and soccer players [3]. Disruption of this ligament is three to ten times more common in female athletes than in male athletes [4].

The annual incidence of ACL injury is estimated at about 1 in 3,500 among the general population [3]. If it is left untreated, there is a significantly higher risk of functional instability, subsequent meniscus tears, and osteoarthritis in the knee [5]. Therefore, it is essential to treat ACL injury. The conventional treatment for ACL injury for patients who want to return to sports is ACL reconstruction [6].

Both surgical and non-surgical treatment approaches are appropriate after ACL injury, and the choice of treatment depends on concurrent injuries, risk factors, degree of activity, and the patient's expectations and goals [7]. The main management options for ACL injury as first-line treatment are rehabilitation (followed by ACL reconstruction in patients, who develop functional instability), ACL reconstruction and post-operative rehabilitation, and pre-operative rehabilitation followed by ACL reconstruction and post-operative rehabilitation [8].

ACL reconstruction surgery involves replacing the injured ACL with an autograft (tissue removed from the person's own body, such as a hamstring tendon), an allograft (ligament extracted from a human cadaver, or a properly treated tendon), or synthetic graft, under arthroscopic management [9,10]. The most suitable graft for ACL reconstruction is one that is biomechanically equivalent to native ligament, is easily harvested, has the least amount of harvest site morbidity, can be secured predictably, and integrates well with bone [10].

In recent years, adjustable-loop devices are the newer version of femoral cortical suspension devices, as they are simple to use, provide excellent results, and offer multiple advantages. One of the most commonly used devices is the titanium adjustable loop button that was used for soft-tissue fixation to the bone since titanium is thought to be the most biocompatible metal, due to its bio-inertness, corrosion resistance, high fatigue limit, and capacity for osseointegration [11,12,13]. Polyetheretherketone (PEEK) materials are stable and biocompatible and are utilized in orthopedic surgery [14]. In ACL reconstruction, an adjustable loop button is used along with the PEEK button to provide better postoperative imaging and stable fixation benefits. The all-inside approach has become an effective option for treating meniscal injuries because of reduced operative times and quicker patient recovery [15].

After ACL reconstruction surgery, patients' functional outcomes, for instance, knee stability, pain levels, and capacity to return to physical activities are crucial indicators of the success of the procedure [16].

The present study hypothesized that the Proloop^TM^-Titanium adjustable loop button and T-Button® A closed PEEK button (Healthium Medtech Limited, Bengaluru, Karnataka, India) used for ACL reconstruction should be able to produce a high rate of stability with a low complication rate among reconstructed knees. Therefore, the study aims to assess the functional outcomes and safety associated with the Sironix® titanium button and PEEK button when used for ACL reconstruction.

## Materials and methods

Device description

Proloop-Titanium Adjustable Loop Button

**Figure 1:**
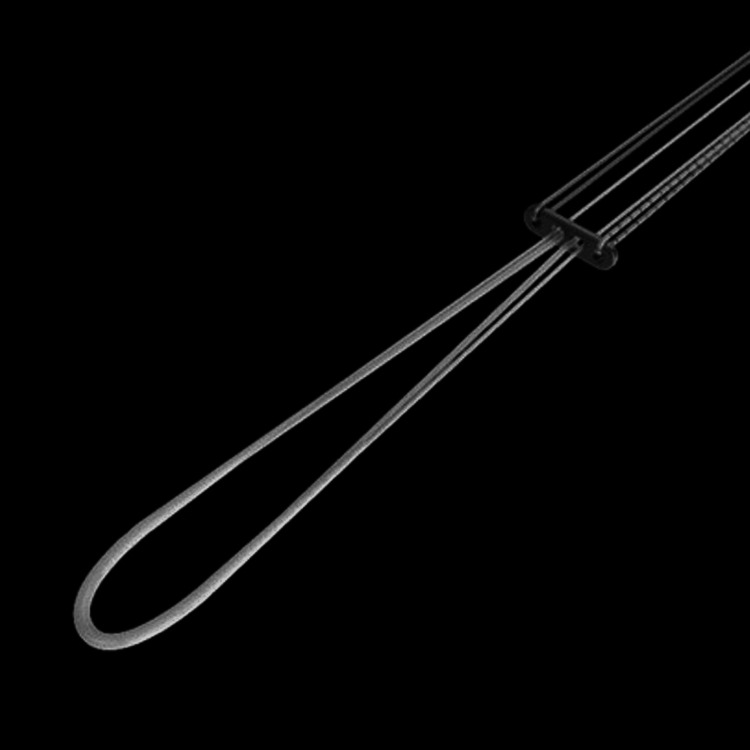
Proloop-Titanium adjustable loop button (Healthium Medtech Limited, Bengaluru, Karnataka, India)

The titanium adjustable loop button is made up of two components: a variable suture loop and a metal fixation device (i.e., button). The suture portion of the fixation device is made of a UHMWPE (ultrahigh-molecular-weight polyethylene). The fixation device (button) is made of a titanium alloy. It is intended for soft tissue fixation to the bone (Figure 1).

T-Button A Closed PEEK Button

**Figure 2:**
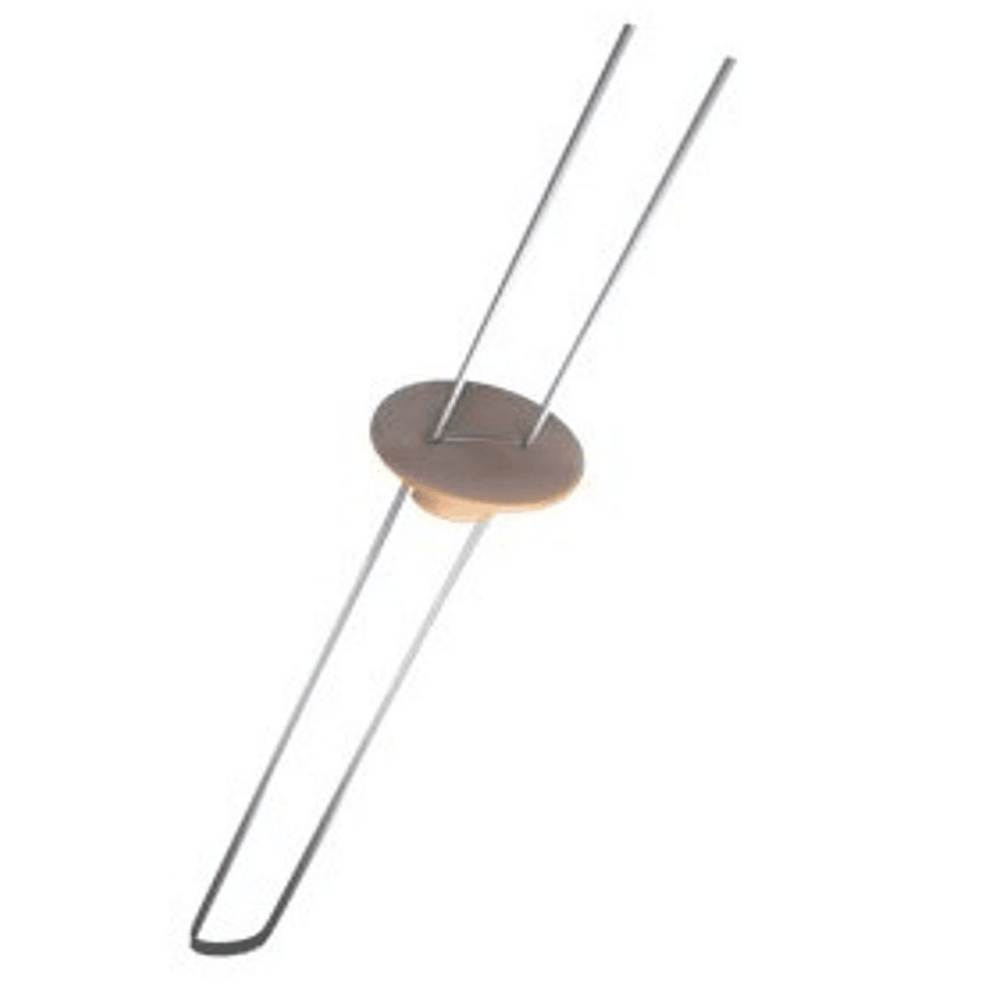
T-Button® A closed PEEK button (Healthium Medtech Limited, Bengaluru, Karnataka, India)

The T-Button A adjustable loop UHMWPE suture closed PEEK button is made up of two components: a variable suture loop and a PEEK fixation device (i.e., button). The suture portion of the fixation device is made of a UHMWPE. The fixation device (button) is made up of PEEK. It is intended for soft tissue fixation to the bone (Figure 2).

Surestitch^TM^ All Inside Meniscal Repair Implant

**Figure 3:**
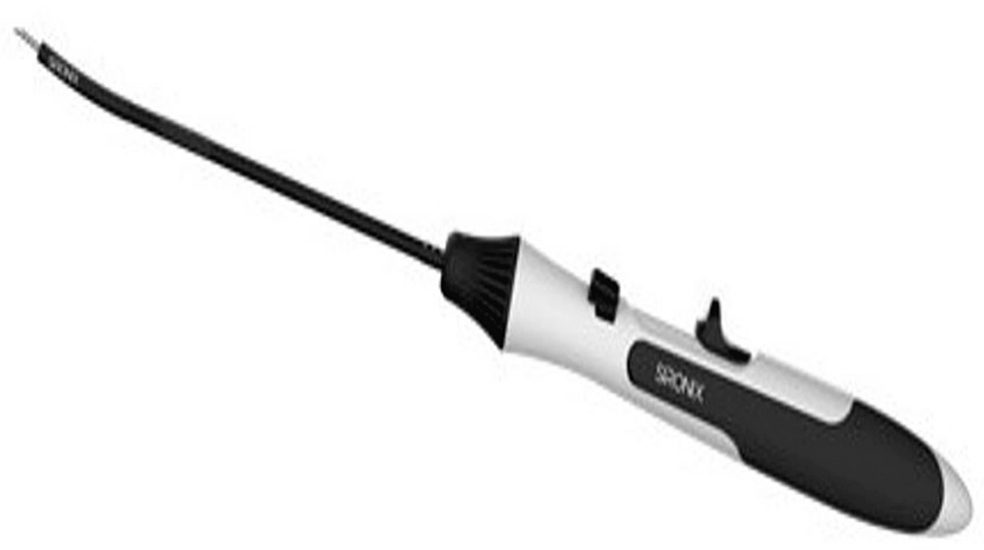
Surestitch all inside meniscal repair implant (Healthium Medtech Limited, Bengaluru, Karnataka, India)

It comprises PEEK implants, pre-tied with USP #2-0 UHMWPE suture and preloaded into a needle delivery system (Figure 3).

Study design

A retrospective, observational, post-marketing study was performed on patients who underwent ACL reconstruction at the study center (AMRI Hospitals, Bhubaneswar, Odisha, India) between August 2022 and January 2023 using Sironix titanium button and PEEK button of Healthium Medtech Limited. The primary objective of this study was to assess the functional outcomes using the IKDC questionnaire. Secondary objectives include evaluating pre-surgery and post-surgery activity levels using the TAS and Lysholm questionnaires and the quality-of-life subscale of Knee Injury and Osteoarthritis Outcome Score (KOOS) and SANE questionnaires to assess the quality-of-life post-surgery. 

The Institutional Ethics Committee of AMRI Hospitals approved the study. This study was conducted as per the EN ISO 14155:2020 Clinical Investigation of Medical Devices for Human Patients - Good Clinical Practice and ICH E6 (R2) Guideline for Good Clinical Practice (GCP) and MDR 2017 (Medical Device Regulations) as well as applicable local regulations. The clinical trial was registered with the Clinical Trial Registry of India (CTRI) (CTRI/2023/05/052392). A total of 31 subjects (18-60 years) were included in the study. Informed consent was obtained from participants before performing any study-related procedure. Patients who were not able to provide consent and were unwilling to be followed up telephonically or patients with injury to the same knee post-ACL reconstruction procedure were excluded from the study.

Data collection and outcomes

The demographic data, medical history, and ACL reconstruction procedural details of the eligible subjects were recorded from the hospital/site’s in-patients’ medical records. The functional outcome of the knee was evaluated by using five independent assessment scales, namely, the International Knee Documentation Committee (IKDC), Lysholm Knee score, Tegner activity score, KOOS, and Single Assessment Numeric Evaluation (SANE) score.

Statistical analysis

Statistics and descriptive comparisons of the study results were done using SAS version 9.4 (SAS Institute Inc., Cary, NC). Continuous variables were summarized using descriptive statistics such as the number of non-missing observations (n), mean, and standard deviation (SD). For categorical variables, the number of non-missing observations and percentage (%) were calculated for subjects with non-missing data.

## Results

A total of 31 patients (26 males and 5 females) were included in the study. Table 1 shows the patient demographics and ACL reconstruction surgery details.

**Table 1:** Demographic and baseline characteristics *Two devices were used in one subject n: number of patients, %: percentage of patients, SD: standard deviation

Demographics	
Age (years), mean ± SD	31.6 years ± 10.77
Sex, n (%)	
Male	26 (83.9)
Female	5 (16.1)
Height (cm), mean ± SD	170.0 (1.56)
Weight (kg), mean ± SD	73.3 (19.01)
BMI (kg/m^2^), mean ± SD	24.1 (2.24)
ACL reconstruction surgery details	
Side (surgery)	
Right	19 (61.3)
Left	12 (38.7)
Reason for injury	
Severe knee joint pain for more than 1 month	1 (3.2)
Twisting injury	30 (96.8)
Type of injury, n (%)	
ACL injury	19 (61.3)
ACL plus meniscus injury	12 (38.7)
No. of devices used in knee arthroscopy surgery	75
N	31
Mean (SD)	2.3 (0.54)
Type of graft used, n (%)	
Autograft	31 (100.0)
Implant for femoral fixation, n (%)	
Proloop-Titanium adjustable loop button	31 (100.0)
Implant used for tibial fixation	
T-Button A Closed PEEK button	31 (100.0)
Intervention for meniscus Injury n (%)	
Surestitch all inside meniscal repair implant device*	13 (41.9)
Other knee function, n (%)	
Normal	31 (100%)
Abnormal	0

The mean (SD) age, height, and BMI were 31.6 years ± 10.77 years, 170.0 cm ± 1.56 cm, and 24.1 kg/m^2^ ± 2.24 kg/m^2^, respectively. All patients had ACL injuries, of which 19 (61.3) patients had right knee injuries and 12 (38.7) patients had left knee. The reason for injury includes the following: one patient (1 (3.2)) had severe knee joint pain for more than one month and was not aware of any injury, and the remaining 30 (96.8) patients had twisting injuries.

A total of 75 devices were implanted in 31 patients, with the mean (SD) number of devices used in the knee being 2.3 (0.54), including 31 Proloop Titanium Adjustable Loop Buttons, 31 T-Button A Closed PEEK Buttons, and 13 Surestitch All Inside Meniscal Repair Implants. A total of 12 patients underwent surgery on their left knee, while 19 patients had surgery on their right knee. Twelve of 31 subjects had an associated meniscal injury, and meniscus repair was done using Surestitch All inside meniscal repair implants 13 (41.9) (Table 1).

Functional outcomes post ACL reconstruction surgery were evaluated in all subjects using IKDC, Lysholm Knee score, Tegner activity score, KOOS, and SANE score.

Functional outcome measures

Primary Outcome

IKDC score: The mean (SD) value of the IKDC evaluation score for 31 patients was 51.4 (2.84) at baseline and 91.8 (2.59) after surgery. Out of 31 patients, the mean (SD) values of 13 patients with a duration of less than six months post-surgery were 51.3 (2.72) and 91.5 (2.95) at baseline and post-surgery, respectively, while the mean (SD) values of 18 patients with a duration of more than six months post-surgery were 51.4 (3.00) and 92.1 (2.36), respectively (Table 2).

**Table 2:** Subjective IKDC score at baseline and follow-up N: number of patients, SD: standard deviation

Duration		Baseline	Post-Surgery	p-Value
	Total IKDC Score			
	N	31	31	
	Mean (SD)	51.4 ± 2.84	91.8 ± 2.59	0.000
Less than 6 months	Total IKDC Scale			
	N	13	13	
	Mean (SD)	51.3 ± 2.72	91.5 ± 2.95	0.000
More than 6 months	Total IKDC Scale			
	N	18	18	
	Mean (SD)	51.4 ± 3.00	92.1 ± 2.36	0.000

Secondary Outcomes

TAS and Lysholm scores: The Tegner activity scale was used to assess and compare the activity levels of the patients for pre-injury and post-surgery periods. The mean (SD) value of the total Tegner activity scale of 31 patients at pre-injury and post-surgery was 5.3 (1.47) and 5.4 (1.38), respectively. Lysholm Score was used to assess and compare the activity level of the patients for baseline and post-surgery periods. The mean (SD) value of the total Lysholm Score of 31 patients at baseline and post-surgery was 53.9 (3.72) and 91.4 (3.61), respectively (Table 3).

**Table 3:** Subjective Tegner activity scale, Lysholm scale scores at baseline and follow-up N: number of patients, SD: standard deviation

Duration	Baseline Mean (SD)	Post-surgery Mean (SD)	P-Value
Tegner activity scale			
Less than 6 months	5.2 ± 1.34	5.2 ± 1.34	
6 months to 1 year	5.4 ± 1.58	5.5 ± 1.42	0.542
Total	5.3 ± 1.47	5.4 ± 1.38	0.536
Lysholm scale			
Less than 6 months	54.8 ± 3.95	91.2 ± 3.72	0.000
More than 6 months	53.3 ± 3.51	91.6 ± 3.63	0.000
Total	53.9 ± 3.72	91.4 ± 3.61	0.000

KOOS and SANE scores: The quality of life after ACL reconstruction was evaluated using the modified KOOS Quality of Life subscale and Single Assessment Numerical Evaluation (SANE) score. The mean (SD) value of the total KOOS Score (QoL) of 31 patients was 91.2 (3.91) (Table 4).

**Table 4:** Subjective Knee Injury and Osteoarthritis Outcome Score (KOOS) N: number of patients, SD: standard deviation

KOOS Score (QoL)		
Mean (SD)	Less than 6 months (N=13)	91.2 ± 4.16
	More than 6 months (N=18)	91.3 ± 3.85
	Total (N=31)	91.2 ± 3.91

The mean (SD) value of the SANE Score of 31 patients, who had affected joint/region of interest today was 97.4 (1.78), and the opposite side today was 99.5 (0.85) (Table 5).

**Table 5:** Single Assessment Numeric Evaluation (SANE) score N: number of patients, SD: standard deviation

	Less than 6 months (N=13)	6 months to 1 year (N=18)	Total (N=31)
Affected 2 joint/region of interest today mean (SD)	97.8 ± 2.08	97.0 ± 1.50	97.4 ± 1.78
Opposite side today mean (SD)	99.8 ± 0.55	99.3 ± 0.97	99.5 ± 0.85

Adverse events

There were no adverse events reported among the 31 patients. None of the patients were discontinued from the study.

## Discussion

ACL reconstruction surgery is one of the most popular orthopedic procedures performed worldwide. A wide range of grafts and graft fixation methods are used in ACL reconstruction. Arthroscopic ACL reconstruction surgery is extremely effective in achieving knee stability with most of these graft fixation methods. Two elements that contribute to surgical success are the introduction of different types of reliable and powerful graft fixation implants, as well as the development of pre- and post-surgical rehabilitation procedures [17].

Even though there have been many studies comparing different graft types, there is no clear consensus on the superiority of one graft over another. However, the majority of the various grafts currently in use are associated with favorable functional outcomes if the proper surgical procedures are performed. In the current study, we obtained good functional outcomes using the Sironix titanium button and PEEK button [17].

In the present study, post-operatively the mean (SD) of the IKDC score was 91.8 ± 2.59 elucidating progress in symptoms, sports activity, and function post-surgery. Similar results were found in a study comparing titanium and PEEK interference screws in which post-operative IKDC scores were 90 ± 8.9 and 89 ± 9.1, respectively, demonstrating no statistically significant differences between both [18]. Another study comparing adjustable-loop and interference screw fixation found that the post-surgery IKDC scores were 75.3 ± 17.4 and 80.5 ± 13.6 in the groups [19].

Lind et al. (2020) demonstrated a study and reported that the mean and standard deviations (SD) of IKDC scores at baseline, six months, and one year after ACL reconstruction were 50.6 (19), 70.8 (1.6), and 73.7 (1.9), respectively. The current study results revealed that the IKDC scores at baseline and less than six months were 51.3 (2.72) and 91.5 (2.95), respectively. The IKDC scores at baseline and more than six months were 51.4 (3.00) and 92.1 (2.36), respectively [20].

The mean (SD) value of the Tegner activity scale at pre-injury was 5.3 ± 1.47 and post-surgery was 5.4 ± 1.38, indicating a substantial return to pre-injury activity levels in the recreation, daily living, and competitive sports. In a study comparing functional outcomes in the isolated posterior cruciate ligament (PCL) group and the combined ACL and PCL group with titanium interference screws equivalent results were found. In TAS at pre-surgery to post-surgery at five years of follow-up in both groups were 6.83 to 6.23 and 6.72 to 5.82, respectively [21].

The present study results revealed that the total mean (SD) value of the Lysholm Score at baseline and post-surgery was 53.9 ± 3.72 and 91.4 ± 3.61, respectively. Comparable results were found in a study showing stability results of Hamstring ACL reconstruction using Endobutton, in which the post-surgery Lysholm score was 90 ± 12.16 [22]. Another study revealed post-operative Lysholm scores in the groups comparing clinical results and tunnel widening following hamstring ACL reconstructions with fixed- and adjustable-loop cortical suspension systems, the scores at baseline and post-surgery were 58.3 (16.6), 58.1 (16.2), and 92.6 (9.3), and 94.3 (6.8) [23].

Our study findings found that the mean (SD) of the Lysholm score at baseline and more than six months after ACL reconstruction was 53.3 (3.51) and 91.6 (3.63), respectively. Analogous results were found in a study conducted by Ahn et al. (2020) and reported that the mean (SD) of the Lysholm score at baseline and more than two years after ACL reconstruction was 61.2 (8.6) and 91.0 (6.5), respectively [24].

A favorable outcome with the mean (SD) value of the quality-of-life subscale of the KOOS score obtained was 91.2 ±3.91, representing improved quality-of-life post-surgery. Similar results found in a study that showed improved quality of life in patients post ACL reconstruction was 81.8 [25]. Our research revealed that the KOOS score for more than six months was 91.3 (3.85). This result was in contrast to a study by Hill et al. assessed the mean (SD) of KOOS (QoL) score for one year after ACL reconstruction surgery as 78.1 (20.2) [26].

In the current study, the overall mean (SD) value of the SANE score of 31 patients who had affected joint/region of interest today was 97.4 (1.78), and the opposite side today was 99.5 (0.85). Similar results were found in a retrospective observational study in which the average SANE score of the patients, who had affected joint/region of interest today was 81% ± 11, and the opposite side today was 100% [27].

Limitations

The present study has a few limitations. First, as a retrospective study, more prospective studies including randomized controlled trials should be performed to provide stronger evidence. Second, the study sample size is quite small. However, follow-up data in this study generated in a real-world setting add value. The study's significant correlation with existing literature supports the findings, as evidenced by the IKDC score, SANE score, Lysholm score, Tegner Activity Scale, and KOOS quality of life score.

## Conclusions

Patients who underwent ACL reconstruction with the Sironix study implants experienced notable improvement in their knee functionality. This was evident through a substantial increase in functional assessment scores (including IKDC, Lysholm, TAS, KOOS, and SANE) observed during the follow-up period. Consequently, the findings of the study support the hypothesis that employing Sironix knee implants (comprising Proloop-Titanium adjustable loop button, T-Button A closed PEEK button, and Surestitch all Inside meniscal repair implant) for ACL reconstruction and meniscal repair is both safe and effective, yielding positive functional results.

## References

[1] McLean SG, Mallett KF, Arruda EM (2015). Deconstructing the anterior cruciate ligament: what we know and do not know about function, material properties, and injury mechanics. J Biomech Eng.

[2] LaBella CR, Hennrikus W, Hewett TE (2014). Anterior cruciate ligament injuries: diagnosis, treatment, and prevention. Pediatrics.

[3] Evans J, Nielson J (2023). Anterior cruciate ligament knee injuries. StatPearls [Internet].

[4] Slauterbeck JR, Hickox JR, Beynnon B, Hardy DM (2006). Anterior cruciate ligament biology and its relationship to injury forces. Orthop Clin North Am.

[5] Hagino T, Ochiai S, Senga S, Yamashita T, Wako M, Ando T, Haro H (2015). Meniscal tears associated with anterior cruciate ligament injury. Arch Orthop Trauma Surg.

[6] Satora W, Królikowska A, Czamara A, Reichert P (2017). Synthetic grafts in the treatment of ruptured anterior cruciate ligament of the knee joint. Polim Med.

[7] Diermeier TA, Rothrauff BB, Engebretsen L (2021). Treatment after ACL injury: Panther Symposium ACL Treatment Consensus Group. Br J Sports Med.

[8] Filbay SR, Grindem H (2019). Evidence-based recommendations for the management of anterior cruciate ligament (ACL) rupture. Best Pract Res Clin Rheumatol.

[9] Zeng C, Lei G, Gao S, Luo W (2018). Methods and devices for graft fixation in anterior cruciate ligament reconstruction. Cochrane Database Syst Rev.

[10] Dhammi IK, Rehan-Ul-Haq Rehan-Ul-Haq, Kumar S (2015). Graft choices for anterior cruciate ligament reconstruction. Indian J Orthop.

[11] Sharma P, Baghel A, Keshav K, Kumar A, Singh A, Singh AB (2023). Functional outcomes of anterior cruciate ligament reconstruction using titanium adjustable loop button and poly-L-co-DL-lactic acid-beta tricalcium phosphate (PLDLA-BTCP) interference screws: a single-center, retrospective, observational study. Cureus.

[12] Khan MJ, Asif N, Aziz MH (2022). Does an adjustable-loop device loosen following ACL reconstruction with a hamstring graft? A retrospective study with a follow-up of two years. J Clin Med.

[13] Kim Y, Kubota M, Muramoto K (2021). Clinical and radiographic results after ACL reconstruction using an adjustable-loop device. Asia Pac J Sports Med Arthrosc Rehabil Technol.

[14] Gao P, Yuan M, Xu Y (2022). The safety and effectiveness comparison of Delta Medical's PEEK interface screw and Endobutton and that of Smith & Nephew's in arthroscopic anterior cruciate ligament reconstruction: a multicenter prospective double-blind randomized controlled clinical trial. Front Public Health.

[15] Ardizzone CA, Houck DA, McCartney DW, Vidal AF, Frank RM (2020). All-inside repair of bucket-handle meniscal tears: clinical outcomes and prognostic factors. Am J Sports Med.

[16] Paschos NK, Howell SM (2017). Anterior cruciate ligament reconstruction: principles of treatment. EFORT Open Rev.

[17] Türkmen F, Basbug V, Özer M, Kesik K, Kacıra B (2021). Clinical comparison of transfix and tightrope fixations in patients with arthroscopic anterior cruciate ligament reconstruction.

[18] Shumborski S, Heath E, Salmon LJ, Roe JP, Linklater JP, Facek M, Pinczewski LA (2019). A randomized controlled trial of peek versus titanium interference screws for anterior cruciate ligament reconstruction with 2-year follow-up. Am J Sports Med.

[19] Lee TJ, Jang KM, Kim TJ, Lee SM, Bae JH (2022). Adjustable-loop cortical suspensory fixation results in greater tibial tunnel widening compared to interference screw fixation in primary anterior cruciate ligament reconstruction. Medicina (Kaunas).

[20] Lind M, Nielsen T, Sørensen OG, Mygind-Klavsen B, Faunø P, Leake-Gardner S (2020). Bone ingrowth into open architecture PEEK interference screw after ACL reconstruction. J Exp Orthop.

[21] Gupta R, Singhal A, Kapoor A, David Masih G, Jhatiwal S (2021). Similar functional outcomes of arthroscopic reconstruction in patients with isolated posterior cruciate ligament (PCL) and combined anterior cruciate ligament (ACL) and PCL tears. J Clin Orthop Trauma.

[22] Prodromos CC, Han YS, Keller BL, Bolyard RJ (2005). Stability results of hamstring anterior cruciate ligament reconstruction at 2- to 8-year follow-up. Arthroscopy.

[23] Choi NH, Yang BS, Victoroff BN (2017). Clinical and radiological outcomes after hamstring anterior cruciate ligament reconstructions: comparison between fixed-loop and adjustable-loop cortical suspension devices. Am J Sports Med.

[24] Chen K, Zhu W, Zheng Y (2020). A retrospective study to compare the clinical effects of individualized anatomic single- and double-bundle anterior cruciate ligament reconstruction surgery. Sci Rep.

[25] Davis-Wilson HC, Thoma LM, Longobardi L, Franz JR, Blackburn JT, Hackney AC, Pietrosimone B (2022). Association of quality of life with moderate-to-vigorous physical activity after anterior cruciate ligament reconstruction. J Athl Train.

[26] Hill GN, O'Leary ST (2013). Anterior cruciate ligament reconstruction: the short-term recovery using the Knee Injury and Osteoarthritis Outcome Score (KOOS). Knee Surg Sports Traumatol Arthrosc.

[27] Yathiraj BR, Iyengar SS, Moharana AK, Angrish S, Deepak TS (2023). Functional outcomes following arthroscopic ACL reconstruction with fixed loop suture button and interference screw: a retrospective observational study. J Orthop Trauma Surg.

